# Assessing the Needs of Elderly People in a Home Environment: Perspectives from Patients, Caregivers, and a Family Nurse

**DOI:** 10.3390/healthcare13080860

**Published:** 2025-04-09

**Authors:** Marlena Szewczyczak, Dorota Talarska, Magdalena Strugała, Patrycja Talarska-Kulczyk, Kamila Kawecka, Katarzyna Wieczorowska-Tobis, Sławomir Tobis

**Affiliations:** 1 Department of Preventive Medicine, Faculty of Health Sciences, University of Medical Sciences, 60-608 Poznań, Poland; mszewczyczak@ump.edu.pl (M.S.);; 2 Department of Immunobiology, Poznań University of Medical Sciences, 60-608 Poznań, Poland; 3 Laboratory for Geriatrics, Department of Palliative Medicine, University of Medical Sciences, 60-608 Poznań, Poland; kwt@tobis.pl; 4 Department of Human Nutrition and Dietetics, Poznan University of Life Sciences, 60-624 Poznań, Poland; 5 Occupational Therapy Unit, Faculty of Health Sciences, University of Medical Sciences, 60-608 Poznań, Poland

**Keywords:** needs, elderly people, home environment, Camberwell Assessment of Need for the Elderly (CANE)

## Abstract

**Background:** Social demographic changes contribute to increased life expectancy and disability. The ability to maintain independence depends on receiving appropriate support. This study aimed to analyze the needs of individuals over 75 years of age living in a home environment. **Methods:** A cross-sectional study assessed support needs from the perspectives of the patient, caregiver, and researcher. The following research tools were used: Camberwell Assessment of Need for the Elderly (CANE), Mini-Mental State Examination (MMSE), Barthel Index, and Geriatric Depression Scale (GDS). **Results:** The average total number of needs reported by the patients was 5.0 ± 2.9, by caregivers 6.63 ± 2.98, and by researchers 5.76 ± 3.43. The most frequently reported unmet needs were related to Accommodation, Company, and Eyesight/Hearing/Communication. A higher number of met needs (*p* = 0.006) and total needs (*p* = 0.011) was observed in individuals aged 85 and older and in seniors who had a caregiver (*p* < 0.001). Lower functional ability was an indicator of a higher number of met needs (*p* < 0.001). Cognitive function did not affect the number of reported needs. A correlation was found between the number of needs and depressive symptoms. The total number of needs reported by patients was significantly lower than the number reported by healthcare personnel and caregivers. **Conclusions:** The CANE questionnaire enabled the identification of individual care needs in the elderly. Although unmet needs were in areas that could be easily addressed, elderly individuals did not receive adequate support. Researchers and caregivers identified more met and unmet needs than the elderly individuals themselves.

## 1. Introduction

Access to healthcare and improved service quality significantly contribute to the extension of life expectancy worldwide [1,2]. By 2050, individuals aged 80 and over are projected to constitute 16% of the global population [3]. Unfortunately, increased longevity does not always correlate with sustained physical and mental fitness [2,3,4]. Aging is often accompanied by disease processes that significantly impair the body’s vital functions. In old age, the most common conditions include hearing loss, cataracts, back and neck pain, osteoarthritis, chronic obstructive pulmonary disease, diabetes, depression, and dementia. Additionally, multimorbidity is becoming more frequent [2,5,6,7]. Health changes affect the ability to perform basic and instrumental activities of daily living (ADL, IADL) [5]. Functional limitations increase the demand for caregiving support [5]. In the United Kingdom, the number of elderly individuals requiring caregiving support is expected to rise by one-third by 2035. Currently, 20% of older men and 30% of older women require assistance with basic daily activities [4]. In Poland, as of 2023, 26.3% of the total population was aged 60 or older [8]. Severe and moderate limitations in daily activities (lasting at least six months) affected 45.1% of the elderly individuals, with the percentage being 4.4% higher among women than men. The primary causes of disability were diseases and health disorders [8].

In many countries, caregiving support is organized by families in cooperation with state institutions (such as social care services) or community organizations. The problem intensifies when families cannot provide care due to work commitments, health conditions, or the need to care for another family member [5,6].

A 2023 report from the Polish Ministry of Senior Policy indicated that the overall population decline and the increasing number of elderly individuals have led to a higher dependency ratio (the number of people aged 65+ per 100 individuals aged 15–64), reaching 30.9 in 2023, a trend observed since 2011 [8]. Caregiving support imposes a significant financial burden on the state. Therefore, the main goal of senior care planning should be to maintain the longest possible functional independence and enable elderly individuals to live independently in their own homes [1,3]. Well-being in old age is influenced by physical, mental, and cognitive health, participation in social life, ability to perform daily activities (ADLs), and financial independence [1,3]. Unmet care needs are defined as problems that have not been adequately addressed, or for which appropriate action has not been taken, leading to persistent issues [9,10,11]. The most frequently unmet needs among the elderly include physiological, psycho-emotional, spiritual, economic, infrastructural, transportation, and educational/informational needs [1,9]. Properly addressing these problems can facilitate independent living despite impairments, while unmet needs can worsen health conditions, resulting in hospitalization or institutional care, which increases healthcare costs [7,9,10,11].

Needs are perceived differently depending on who is assessing them. Patients, informal caregivers, medical or social care workers, and independent researchers evaluate needs from different perspectives, which affects how they are interpreted [7,9,11].

A reliable assessment tool is necessary to facilitate accurate need identification, ensuring that needs are consistently reported regardless of the assessor. The Camberwell Assessment of Need for the Elderly (CANE) questionnaire is one such tool [11,12,13].

CANE assesses needs from three perspectives, the patient, caregiver, and healthcare personnel, within 30 min [9,11,12,14]. It has strong psychometric properties, including reliability (Cronbach’s α = 0.99) and validity (correlated with Index Barthel r = −0.53; CAPE-BRS r = 0.66). The concordance between the assessment of needs from the perspective of the patient and that of the researcher was (Cohen’s kappa) 0.90, while between the staff and the caregiver, it was 0.98, and between the patient and the caregiver, it was 0.89 [15]. Following a brief training session, CANE can be used by various healthcare professionals and provides insights into caregivers’ needs as well [7,11,12,16].

Originally developed for individuals with mental illnesses, CANE is also applicable to patients with somatic diseases and in long-term care settings [7,11,12,16]. A key advantage of CANE is that it allows comparison of patient-reported needs with caregiver and staff assessments, enabling verification of the effectiveness of provided support.

This study aimed to compare the perceived needs of elderly individuals living at home with those of their caregivers and a family nurse. This is one of the first studies in Poland evaluating the needs of home-dwelling elderly individuals from three perspectives. In a review of the literature, there were a few studies that considered elderly individuals living in a home environment without mental disorders [7,12,17].

## 2. Materials and Methods

The cross-sectional study included 200 randomly selected individuals who had registered with a Primary Healthcare Nurse and were at least 75 years old. A total of 483 surveys were included in the final analysis, comprising 193 surveys completed by the family nurse (researcher), 193 by the patients, and 97 by the caregivers. Seven surveys were incorrectly completed.

Inclusion criteria: Patient and caregiver consent to participate in the study; registration with a family nurse; living in a home environment; age ≥ 75 years; and a cognitive function level allowing the patient to understand the questions (MMSE > 15 points).

Exclusion criteria: Lack of consent from the patient or caregiver to participate; age < 75 years; low cognitive function level (MMSE < 15 points); severe advanced somatic diseases preventing participation in the study; and palliative care status.

This study was approved by the Bioethics Committee at the Medical University of Karol Marcinkowski in Poznań, Poland. The Commission also confirmed that the study did not bear the characteristics of a medical experiment (date: 19 December 2018; no. KB 537/24). The research was conducted in accordance with the principles of the Declaration of Helsinki, developed by the World Medical Association, outlining the ethical principles for conducting medical research with the participation of humans.

### 2.1. Research Organization

Structured face-to-face interviews were conducted by a trained family nurse during home visits with randomly selected patients between 2022 and 2023. The training for all individuals participating in the study was facilitated by K. Wieczorowska-Tobis, who had previously conducted research using the CANE questionnaire. Before each interview, the nurse explained the study’s purpose and described the assessment tools. Verbal informed consent was obtained from each patient and caregiver before inclusion in the study (a single nurse conducted the study).

### 2.2. Research Tools

1. The Mini-Mental State Examination (MMSE) is a brief screening tool used to identify individuals at an increased risk of dementia [18]. The scoring range is from 0 to 30, where a score of 27–30 indicates normal cognitive function, 24–26 suggests mild cognitive impairment without dementia, 20–23 corresponds to mild dementia, and 15–19 indicates moderate dementia. The obtained scores were adjusted for age and education level [19].
2. The Barthel Index is used to assess independence in performing basic activities of daily living (ADLs) [20,21]. The scale ranges from 0 to 100 points, with the following three categories commonly used in Poland: 0–20 points indicate complete dependence, 21–79 points suggest the patient requires assistance in some activities (moderate-to-severe impairment), and 80–100 points signify mostly independent functioning with minimal assistance (mild impairment). A score of 0–40 points qualify a patient for long-term care services.
3. The Geriatric Depression Scale (GDS) is a screening tool used in comprehensive geriatric assessments to help diagnose depression in older adults [22]. The scoring range is from 0 to 15, with a cut-off point of > 5 indicating depression. The severity of depression is classified as 6–9 points for mild depression and 10–15 points for moderate-to-severe depression [23,24].
4. The Camberwell Assessment of Need for the Elderly (CANE) is a comprehensive tool developed to assess the care needs of older adults [6,7,11,13,14,17,25,26]. The Polish version of CANE, validated in a pilot study, demonstrated good psychometric properties [27]. Cronbach’s alpha coefficient for the needs assessment in the patient group was 0.857, and for the researcher, it was 0.841. Cohen’s kappa coefficient for the entire questionnaire was 0.864 [27].

The questionnaire evaluates 24 areas grouped into four key dimensions. The Physical Dimension includes Physical health, Medication, Eyesight/Hearing/Communication, Mobility/Falls, Self-Care, and Continence. The Environmental Dimension covers Accommodation, Household Skills, Food, Caring for Others, Money, and Benefits. The Social Dimension focuses on Company, Intimate Relationships, Daytime Activities, Information, and Abuse/Neglect. The Psychological Dimension assesses Psychological Distress, Memory, Inadvertent Self-Harm, Deliberate Self-Harm, Behavior, Psychotic Symptoms, and Alcohol [11,14].

The CANE questionnaire uses a three-level scoring system, where 0 = No Need, 1 = Met Need (a problem that has received appropriate intervention), and 2 = Unmet Need (a problem that remains without proper intervention). For each participant, the number of met and unmet needs was calculated, as well as the total number of needs, which was the sum of both the met and unmet needs [7,11,13,14,15,16,25,28].

Demographic data were collected using a survey incorporated into the CANE questionnaire’s structure.

### 2.3. Statistical Analysis

Statistical analysis was performed using STATISTICA 13.0 (StatSoft, Kraków, Poland) and SPSS (IBM, Warsaw, Poland, https://www.ibm.com/spss). The mean and standard deviation (mean ± standard deviation) were calculated for all analyzed variables. The Shapiro–Wilk test was used to assess the normality of distribution. Since most variables did not follow a normal distribution, the median (M) and data range (Z) were also calculated. Based on the responses obtained from the CANE questionnaire, the number of met and unmet needs was calculated for each participant, and the total number of needs was the sum of the two. The analysis considered the perspectives of the patient, healthcare personnel (family nurse), and caregiver, if applicable. The Mann–Whitney U test was used for independent group comparisons, with *p*-values <0.05 considered statistically significant. Cohen’s kappa coefficient was calculated for two-way comparisons (patient vs. family nurse, patient vs. caregiver) to assess the agreement between the different perspectives. In contrast, Fleiss’ kappa coefficient was used for three-way comparisons (patient vs. family nurse vs. caregiver). Since the number of needs did not follow a normal distribution among the patients, healthcare personnel, and caregivers, the Wilcoxon signed-rank test was used to compare the number of needs across different perspectives.

## 3. Results

### 3.1. Characteristics of the Study Group

The analysis included data from 193 patients, of whom 162 were women (83.94%). The mean age of the respondents was 83.9 ± 5.6 years, with women averaging 84.1 ± 5.7 years and men 82.9 ± 5.3 years, showing comparable age distribution between the genders. The mean Barthel Index score for all participants was 83.9 ± 16.6 points, the mean MMSE score was 24.5 ± 3.3 points, and the mean GDS score was 4.8 ± 2.6 points. Only eight patients (4.15%) met the Polish eligibility criteria for long-term care, defined as a Barthel Index score of ≤40 points (Table 1).

The study involved 97 caregivers, with an average age of 61 ± 5.6 years. Forty (41.24%) caregivers were professionally active. In the study group, women fulfilled the role of caregivers solely. All of them lived with the elderly individuals included in the study.

### 3.2. Needs Analysis from the Patients’ Perspective

The mean total number of needs in the study group was 5.00 ± 2.92, with the majority being met needs (4.43 ± 2.70), while the mean number of unmet needs was 0.57 ± 1.04. The most frequently reported met needs were related to Physical health (79.6%), Mobility/Falls (60.2%), and Household skills (59.1%). In contrast, the most commonly reported unmet needs were in the areas of Accommodation (11.8%), Company (9.7%), Eyesight/ Hearing/ Communication (7.5%), and Psychological Distress (5.4%). The total number of needs was significantly higher in the age group of 85 years and older compared to those aged 75–84 years (*p* < 0.01; Table 2).

The total number of needs was significantly higher in patients whose functional status was classified as moderate-to-severe impairment, according to the Barthel Index, compared to those with mild impairment (*p* < 0.001, Table 2). A similar relationship was observed for the met needs (*p* < 0.001). Among individuals with moderate-to-severe impairment, the most frequently met needs were related to Household Skills (90.2%), Mobility/Falls (85.4%), and Physical Health (82.9%). The highest proportion of unmet needs in this group was in the Company domain (12.2%).

In contrast, among individuals with mild functional impairment, the most commonly met needs were related to Physical Health (76.9%), while unmet needs were most frequently reported in Accommodation (15.4%). When considering depressive symptoms based on the GDS scale, the total number of needs was significantly higher in individuals with at least six points on the GDS, compared to those without depression (*p* < 0.001, Table 2). A similar trend was observed for both the met (*p* < 0.021) and unmet needs (*p* < 0.001). The number of needs reported by individuals with and without dementia (MMSE ≤ 23 points) was comparable. However, among the individuals with dementia, the highest number of unmet needs was reported in the areas of Accommodation (14.3%) and Company (11.4%). In contrast, among those without dementia, the most frequently unmet needs were Accommodation (10.3%), as well as Eyesight/Hearing/Communication and Company (both 8.6%).

### 3.3. Needs Analysis from the Researcher’s Perspective

According to the family nurse conducting the study, the mean total number of needs in the study group was 5.76 ± 3.43, with the majority being met needs (4.83 ± 2.70). The mean number of unmet needs was 0.94 ± 1.68. From the nurses’ perspective, the most frequently met needs were related to Physical Health (87.1%), Household Skills (63.4%), and Mobility/Falls (60.2%). In contrast, the highest proportion of unmet needs was observed in the areas of Company (16.3%), Eyesight/Hearing/Communication (15.1%), and Accommodation (12.9%).

### 3.4. Needs Analysis from the Caregivers’ Perspective

From the caregivers’ perspective, the mean total number of needs in the study group was 6.63 ± 2.98, with the majority being met needs (6.06 ± 2.62). The mean number of unmet needs was 0.57 ± 0.87. According to the caregivers, the most frequently met needs were related to Physical Health (91.7%), Household Skills (81.6%), and Mobility/Falls (73.5%). The highest proportion of unmet needs, as observed by the caregivers, was in the domain of Company (14.3%).

### 3.5. Agreement Between Perspectives

To determine the agreement of the needs assessments between the subjects and staff, as well as between the subjects and caregivers, the percentage agreement between the responses and Cohen’s kappa coefficients was calculated. The calculated average Cohen’s kappa coefficients showed moderate agreement both between patients and staff—0.69 (range 0.00–1.00) and between subjects and caregivers—0.72 (range 0.00–1.00). In the assessment of subjects and family nurse (researcher), high agreement was found in the areas of Household Skills, Food, Eyesight /Hearing/Communication, and Mobility/Falls. Weak agreement was noted in the domains of Physical Health, Information about health status and treatment, and Intimate Relationships. Between the subject and caregiver, the greatest agreement was found in the areas of Abuse/Neglect (1.0), Food (0.92), Medication (0.83), and Household Skills (0.82). Low agreement of assessments between caregivers and subjects was found in the domains of Physical Health (0.31), Deliberate Self-Harm (0.49), and Information about health status and treatment (0.56).

In the study group, the total number of needs was statistically significantly lower than the total number of needs reported by the researchers (T = 159.5; *p* < 0.001) and caregivers (T = 80.5; *p* = 0.027). The number of unmet needs was statistically significantly higher in the group of researchers than in the group of subjects (T = 57, *p* = 0.005). Similarly, the number of met needs was statistically significantly higher in the researchers’ group (T = 48, *p* = 0.01). The number of met needs was statistically significantly higher in the caregivers’ group than the subjects ‘group (T = 107.00; *p* = 0.048).

Fleiss’ kappa coefficient was used to assess the agreement of all three perspectives (Table 3). The average value of Fleiss’ kappa coefficient for the assessments of the subject, researcher, and caregiver was 0.57, indicating moderate agreement. Agreement in individual domains ranged from 0.43 (Physical health) to 0.75 (Money). The greatest agreement was found in the areas of Money, Food, Medication, and Mobility/Falls.

## 4. Discussion

Depending on the nature and degree of difficulty in independent functioning, elderly individuals require different levels of caregiving support [2,9,12,17,29]. In our study, patients reported an average of 5 needs, including 4.43 needs met and 0.57 unmet. In studies conducted by other authors, the average number of unmet needs for individuals living in home environments ranged from 0.3 to 3.3, and the number of needs met ranged from 3.5 to 10.0 [11]. The differences are likely due to the selection of the group, particularly the health condition of the individuals involved in the study. In studies where authors used the CANE questionnaire, patients with dementia or other mental disorders are most often included. People with mental disorders, regardless of their living environment (home, nursing home, hospital), generally report more needs than cognitively functional individuals [6,13,14,25,28]. In our own study, individuals with dementia reported a comparable number of met and unmet needs to those with normal cognitive functions. The difference between the results obtained by the other authors and those in our study may be due to the disproportionality of the group, as the majority of participants in our study had no dementia or mild dementia, and the functional status of the entire group was comparable.

Similar to the studies by other authors, in our study, more needs were reported by individuals in the older age group with lower functional abilities (Barthel Index) and those with symptoms of depression (GDS ≥6) [5,6,7,17,28]. Unmet needs were more common among those who felt lonely, experienced higher levels of anxiety, and had lower social engagement [7].

The elderly participants in our study most frequently reported unmet needs in the domains of Accommodation, Company, Eyesight/Hearing/Communication, and Psychological Stress. In a study conducted by other authors among patients in home environments, the most common unmet needs related to the domains of Physical Health, Information about health status and treatment, Mobility/Falls, and Eyesight/Hearing/Communication [7]. The differences may arise from different living conditions and psycho-physical status. The elderly participants in our study demonstrated good performance in basic activities of daily living. However, they lived in urban tenement buildings and multi-apartment houses without elevators and municipal heating. Therefore, most of them indicated inappropriate living conditions. In addition, the lack of an elevator and a permanent caregiver made it difficult to interact with others. This may also explain the feelings of loneliness and the development of depression in approximately 37% of the participants in our study. A domain where elderly individuals frequently report unmet needs, regardless of the study setting, is Eyesight/Hearing/Communication [6,7,16,17,25]. The results indicate that, similar to our study, the elderly groups in the studies by other authors did not receive sufficient support in this area. Perhaps elderly individuals and caregivers treat the deterioration of vision and hearing as part of the natural aging process, so they do not take appropriate actions to improve hearing and vision. Impaired eyesight and hearing are associated with limitations in mobility, self-care, and household management [29].

The CANE questionnaire used in our study has an additional advantage, as it identifies existing needs and the demand for support from three perspectives, those of the patient, caregiver, and researcher [6,7,11,12,13,14,15,25,26,27]. The authors of earlier studies have pointed out the differences in the reporting of needs between the patient, caregiver, and researcher [7,11,13,15,25]. The researcher and the caregiver may not notice certain needs or assess their importance for the patient differently. An example is a Brazilian study in which the total number of needs reported by participants was 4.9 ± 3.6, with 1.5 ± 1.8 unmet; the caregivers reported 4.7 ± 4.0, with 1.5 ± 2.2 unmet, and the researchers reported 3.8 ± 2.0, with 1.7 ± 2.1 unmet [25]. Awareness of these differences in perceiving needs is particularly important in the case of individuals with dementia or depression, when the individuals under care may be unable to specify their need for support [11,14].

After analyzing the concordance of the reported needs between the patients, the caregivers, and the family nurse in our study, it was found to be at a moderate level (Fleiss’ kappa 0.57). More excellent agreement was found between the patients and caregivers regarding unmet needs. In contrast, further agreement was observed between the patients and the researcher regarding the total number of needs and met needs. Other authors also obtained similar results [7,13,14]. Additionally, in the group of patients with mental disorders, including dementia, it was found that the individuals involved in the study reported fewer met needs than the caregivers and staff. On the other hand, the staff observed more met needs than the caregivers [13,28]. The caregivers reported fewer needs regarding Information about health status and treatment (1.6%) and Psychological Stress (4.0%) [28]. Our study observed the lowest agreement in the Physical Health domains, followed by Behavior and Alcohol. The most excellent agreement was found in the areas of Money and Food. The main nurse (researcher) reported more unmet needs in the Physical Health domain, which may indicate that she noticed problems that the patients and caregivers minimized. In the Behavior domain, the caregivers reported needs more frequently, highlighting patient conflicts.

The knowledge of caregiving needs and reasons for not receiving adequate support facilitates further care planning. One of the reasons for unmet needs is a poorly organized care system, followed by high service costs, the low economic status of society, and low health insurance coverage [10,30]. Additionally, factors associated with service users, such as being an elderly woman, low education, lack of private health insurance, low self-esteem, feelings of depression, and lack of care from close family members or friends, are often mentioned [10,11]. Furthermore, a study conducted in China among patients with memory disorders showed that the total number of needs increased with the patient’s age, the number of years since diagnosis, the MMSE score, and living with someone other than a partner [28]. Meeting caregiving needs is not only important for improving the quality of care but also for the health of the patient. Unmet needs can contribute to health deterioration, leading to higher financial costs for healthcare and social services and a diminished quality of life [7,25].

### Limitations, Strengths, and Future Studies

A valuable element of the study conducted is the presentation of the specific needs of elderly individuals. This knowledge can contribute to the development of support programs focused on identifying and meeting the current needs of older adults. For effective assistance, it is essential to monitor the health and psycho-social situation of elderly individuals and to facilitate cooperation among the various professionals within a geriatric team. Therefore, national social care programs should specify the division of tasks among individual institutions and professionals. It is necessary to include actions such as performing a Comprehensive Geriatric Assessment (CGA) and evaluating caregiving needs, followed by promoting actions for successful aging and preventing the most common disorders, including diseases, loneliness, and falls. Psycho-social activation is also of significant importance. An example of support could be elderly individuals’ participation in senior clubs, daycare centers, or integration activities with younger people, especially children and the youth. Additionally, actions aimed at improving family relationships and the image of elderly individuals are also necessary.

This study also highlights the differences in how the patient and the caregiver perceive needs. Such information sensitizes the staff (nurse, doctor, social caregiver) to the possibility of differences in reporting met and unmet needs and the demand for caregiving support. An additional value of our research is that a single family nurse assessed the needs, which helps to avoid differences in the perception of needs by multiple staff members (researchers).

In the study, it was impossible to perform analyses across all three response dimensions for the entire group due to the absence of a caregiver for every patient. This hindered the comparison of responses from all patients with their caregivers and the comparison of responses from all researchers with caregivers. Therefore, it would be worthwhile to aim for an equal number of patients and caregivers in future studies. It is also valuable to consider the impact of other demographic factors, such as education, and medical factors, such as the number and type of existing health conditions. Repeating the studies regularly and comparing the results obtained is also important. This would provide information on whether the support is provided appropriately, and whether the needs change over a longer period of caregiving. An important aspect that should also be addressed is the needs of the caregivers.

## 5. Conclusions

The CANE questionnaire identifies individual care needs in older adults from three perspectives—the patient, the caregiver, and the researcher. In this study, although the needs pertained to domains where support is relatively easy to provide—such as Accommodation, Company, and Eyesight/Hearing/Communication, as well as Psychological Distress—they were not met. Researchers and caregivers observed more unmet and met needs compared to the older individuals themselves. The caregivers reported the most needs, while the researchers noted the most unmet needs.

## Figures and Tables

**Table 1:** Characteristics of the study group.

Parameter	n (%)
**Age**	
75–84 years	104 (53.89)
≥85 years	89 (46.11)
**Education**	
Primary	73 (37.82)
Vocational	29 (15.03)
Secondary	66 (34.20)
Higher	25 (12.95)
**Marital Status**	
Single	10 (5.18)
Married	54 (27.98)
Informal relationship	4 (2.07)
Divorced/separated	4 (2.07)
Widow(er)	120 (62.18)
**Has a caregiver?**	
Yes	97 (50.26)
No	96 (49.74)

**Table 2:** Characterization of needs based on age, functional ability, cognitive function, and depression severity.

Category	Met Needs (Mean ± SD)	Unmet Needs (Mean ± SD)	Total Needs (Mean ± SD)
**Age**			
75–84 years (N =104)	3.7 ± 2.6	0.6 ± 0.9	4.3 ± 2.9
≥85 years (N = 89)	5.2 ± 2.6	0.6 ± 1.2	5.8 ± 2.8
**Barthel Index**			
21–85 points (N = 85)	5.9 ± 2.4	0.8 ± 1.2	6.7 ± 2.7
86–100 points (N = 108)	3.3 ± 2.4	0.4 ± 0.8	3.7 ± 2.4
**GDS Score**			
0–5 points (N = 121)	3.9 ± 2.5	0.3 ± 0.6	4.2 ± 2.6
6–15 points (N = 72)	5.3 ± 2.8	1.1 ± 1.4	6.4 ± 2.9
**MMSE Score**			
15–23 points (N = 73)	4.5 ± 3.0	0.6 ± 0.8	5.1 ± 3.4
24–30 points (N = 120)	4.4 ± 2.5	0.6 ± 1.2	4.9 ± 2.6

**Table 3:** Agreement of needs assessment in individual areas of the CANE questionnaire according to subjects, family nurse, and caregivers (N = 97).

Area of Needs (CANE)	% Agreement	Fleiss’ Kappa Coefficient
Accommodation	83.7%	0.60
Household skills	89.8%	0.63
Food	89.8%	0.72
Self-care	79.6%	0.61
Care for others	93.9%	0.51
Daytime activities	79.6%	0.58
Memory	87.8%	0.57
Eyesight/Hearing/Communication	75.5%	0.59
Mobility/Falls	85.7%	0.64
Continence	83.7%	0.62
Physical health	83.7%	0.43
Medication	89.8%	0.67
Psychotic symptoms	95.9%	0.53
Psychological stress	85.7%	0.59
Information about health status and treatment	81.6%	0.49
Inadvertent self-harm	98.0%	0.49
Deliberate self-harm	91.8%	0.49
Abuse/Neglect	100.0%	0.54
Behavior	98.0%	0.44
Alcohol	98.0%	0.44
Company	91.8%	0.67
Personal relationships	93.9%	0.48
Money	91.8%	0.75
Benefits	91.8%	0.60

## Data Availability

Data are contained within the article.
